# Does glucose affect the de‐esterification of methyl ferulate by *Lactobacillus buchneri*?

**DOI:** 10.1002/mbo3.971

**Published:** 2019-11-29

**Authors:** Kamyar Mogodiniyai Kasmaei, Dietmar Schlosser, Heike Sträuber, Sabine Kleinsteuber

**Affiliations:** ^1^ Department of Animal Nutrition and management Swedish University of Agricultural Sciences Uppsala Sweden; ^2^ Department of Environmental Microbiology Helmholtz Centre for Environmental Research – UFZ Leipzig Germany

**Keywords:** catabolic repression, feruloyl esterase, fiber digestibility, forage, lignocellulose, silage

## Abstract

Silage, the fermented product from anaerobic storage of forage crops with high water contents (50%–70%), is normally used as animal feed but also for the production of biofuels and value‐added products. To improve the utilization of plant fibers during ensiling, previous attempts have aimed at breaking linkages between lignin and hemicellulose by use of *Lactobacillus buchneri* LN 4017 (ATCC PTA‐6138), a feruloyl esterase (FAE)‐producing strain, but results have been inconsistent. Normally, there are sufficient amounts of readily available substrates for bacterial growth in silage. We thus hypothesized that the inconsistent effect of *L. buchneri* LN 4017 on the digestibility of silage fibers is due to the catabolic repression of FAE activity by substrates present in silage (e.g., glucose). To test this hypothesis, we analyzed the effect of glucose on the de‐esterification of methyl ferulate (MF), a model substrate used for FAE activity assays. At three glucose:MF ratios (0:1, 1:1, and 13:1), the bacteria continued hydrolyzing MF with increasing glucose:MF ratios, indicating that the de‐esterification reaction was not repressed by glucose. We therefore conclude that the de‐esterification activity of *L. buchneri* LN 4017 is not repressed by silage substrates during ensiling.

## Graphical abstract

In this work, we studied de‐esterification activity of *Lactobacillus buchneri* in presence of glucose. Our results indicated that glucose did not repress the de‐esterification reaction. The results obtained provide information about catabolic capacity of *L. buchneri*.

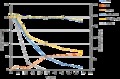

## 1 INTRODUCTION

Plant biomass is considered an important renewable carbon resource with a wide range of applications from animal feeds to feedstock for biorefineries. Production of plant biomass in the form of forage crops (e.g., grass, whole‐crop cereals) is seasonal, and storage is therefore necessary. In temperate regions, anaerobic storage of high water content (50%–70%) forages, known as ensiling, is the most common storage method. During ensiling, epiphytic lactic acid bacteria (LAB) ferment plant sugars to mainly lactic acid, thereby reducing the biomass pH. If anaerobic conditions are maintained, the silage can be stored for several months.

The major parts of plant biomass comprise fibers, which generally have a low digestibility. Plant fibers essentially contain cellulose (30%–55%), hemicellulose (24%–50%), and lignin (12%–35%) (Sharma, Xu, & Qin, 2019). Of these three polymers, only cellulose and hemicellulose can be utilized under anaerobic conditions, for example in the rumen or in anaerobic digesters. In the cell walls of monocots (e.g., cereals and grasses), lignin and hemicellulose are interconnected mainly by ferulic acid (FA), which forms carboxylic ester bonds with arabinose residues of xylan chains on one side and ether bonds with lignin on the other side (Ralph, 2010; Wong, 2006). The lignin–hemicellulose matrix encrusts the cellulose, and this overall configuration results in recalcitrance of plant fibers (Pu, Hu, Huang, Davison, & Ragauskas, 2013; Rubin, 2008). The ester link between FA and hemicelluloses can be cleaved by feruloyl esterases (FAEs) (http://www.chem.qmul.ac.uk/iubmb/enzyme/EC3/1/1/73.html), thereby opening the fiber structure and increasing the bioavailability of fiber constituents for fermentation processes.

Several *Lactobacillus* spp. can produce FAEs (Donaghy, Kelly, & Mckay, 1998), which potentially enables the use of these species as silage inoculants to enhance digestibility of plant fibers during ensiling (Nsereko et al., 2008). Molecular characterization of FAEs from *Lactobacillus* spp. has shown that these enzymes contain a serine active site (https://prosite.expasy.org/PS00120) (Xu, He, Zhang, Guo, & Kong, 2017). The optimal pH of FAEs from *Lactobacillus* spp. varies between 6.5 and 8.0, and the optimal temperature spans a wide range between 20 and 50°C (Esteban‐Torres, Reverón, Mancheño, de las Rivas, & Muñoz, 2013; Fritsch, Jänsch, Ehrmann, Toelstede, & Vogel, 2017; Liu, Bischoff, Anderson, & Rich, 2016; Xu et al., 2017). A recent review comprehensively describes biochemical and molecular properties of microbial FAEs (Oliveira et al., 2019).

*L. buchneri* LN 4017 (ATCC PTA‐6138), a FAE‐producing strain (Nsereko et al., 2008), was used in several studies as the silage inoculant. While fiber digestibility was improved in some cases (Jin et al., 2015; Kang, Adesogan, Kim, & Lee, 2009), no improvement was found in other studies (Kang et al., 2009; Lynch, Baah, & Beauchemin, 2015). One possible explanation for these inconsistent results could be a catabolic repression of FAE activity of the inoculant, caused by the readily available substrates in the silage (e.g., glucose). Such a hypothesis would parallel previous findings with *Aspergillus niger*, where FA induces expression of FAE genes (*faeA* and *faeB*) but fails to induce the expression of these genes in the presence of glucose (de Vries, vanKuyk, Kester, & Visser, 2002). In the present study, we aimed at investigating the effects of varying glucose concentrations on the hydrolytic conversion of methyl ferulate (MF) to FA, a reaction indicative for FAE activity, by *L. buchneri* LN 4017. The ability of *L. buchneri* to grow on the aforementioned compounds was concomitantly assessed.

## 2 MATERIALS AND METHODS

Two experiments were performed. In a primary experiment, de‐esterification of MF by *L. buchneri* LN 4017 (ATCC PTA‐6138) was tested at varying glucose concentrations. To clarify the results obtained, a secondary experiment was conducted to study FA metabolism of this bacterium in the presence of glucose.

De Man, Rogosa and Sharpe (MRS) broth (DSMZ medium 11) without glucose was used as a basal medium. To prepare the inoculum, the bacterium was cultivated anaerobically in MRS broth for 48 hr at 37°C without agitation. Subsequently, 1 ml of bacterial culture was centrifuged at 4,000 *g* for 5 min (21°C). Bacterial cells were thereafter resuspended in 1 ml basal medium and used as inoculum. MF (abcr GmbH) and FA (Merck KGaA) were dissolved in 50% dimethylformamide (DMF) solution (v/v) (Merck KGaA) for medium preparation. The final concentration of DMF in the growth medium was always 0.5% (v/v).

### 2.1 Primary experiment

The following treatments were compared: (a) basal medium containing only 0.5% DMF, (b) basal medium with 199 µg/ml MF, (c) basal medium with 226 µg/ml glucose and 196 µg/ml MF (Glc:MF (1:1)), and (d) basal medium with 2,524 µg/ml glucose and 199 µg/ml MF (Glc:MF (13:1)). Sterile controls were set up for incubations with MF and Glc:MF. All treatments were done in triplicate.

### 2.2 Secondary experiment

The setup included: (a) basal medium with 0.5% DMF, (b) basal medium with 177 µg/ml FA, and (c) basal medium with 2,515 µg/ml glucose and 161 µg/ml FA (Glc:FA). Sterile controls were made for incubations with FA and Glc:FA. All treatments were done in triplicate.

All treatments were incubated anaerobically at 37°C without agitation and were sampled (1 ml) at 0, 4, 8, 12, 24, 36, and 48 hr. Bacterial growth was estimated by measuring optical density at 600 nm. Samples were centrifuged at 20,817 *g* for 10 min (4°C), and supernatants were stored at −20°C until chemical analyses. Upon thawing at room temperature, samples were centrifuged at 20,817 *g* for 20 min (4°C) before analyses for glucose, MF, and FA. Glucose was measured by HPLC as described by Porsch, Wirth, Toth, Schattenberg, and Nikolausz (2015) with the following modifications: operation temperature was 55°C and flow rate was 0.7 ml/min. MF and FA were measured by UPLC according to Hofmann and Schlosser (2016) with the modification of using formic acid for acidification of the mobile phase.

## 3 RESULTS AND DISCUSSION

**Figure 1:**
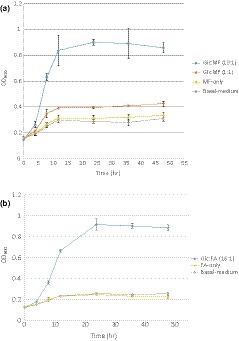
Growth curves of *Lactobacillus buchneri* LN 4017, measured by optical density (OD_600_), when cultivated with: (a) 199 µg/ml methyl ferulate (MF‐only), 226 µg/ml glucose and 196 µg/ml methyl ferulate (Glc:MF (1:1)) and 2,524 µg/ml glucose and 199 µg/ml methyl ferulate (Glc:MF (13:1)) and (b) 177 µg/ml ferulic acid (FA‐only) and 2,515 µg/ml glucose and 161 µg/ml ferulic acid (Glc:FA (16:1)). Mean values of three replicates and standard deviations are shown

In all treatments, bacterial growth reached a stationary phase after 12 hr, except in the Glc:FA treatment, in which the stationary phase was reached after 24 hr (Figure 1). Growth curves were similar for cultures incubated in the basal medium, with MF or with FA.

**Figure 2:**
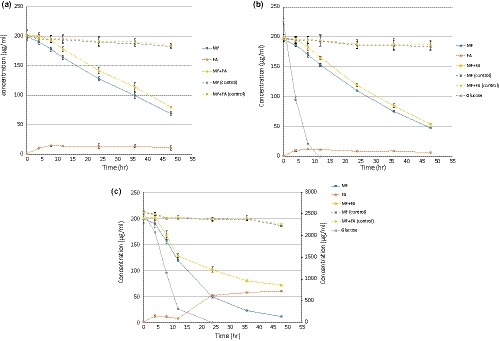
Methyl ferulate (MF) disappearance and ferulic acid (FA) accumulation during cultivation of *Lactobacillus buchneri* LN 4017 with 199 µg/ml MF (a), 226 µg/ml glucose and 196 µg/ml MF (b) and 2,524 µg/ml glucose and 199 µg/ml MF (c). MF (control) and MF + FA (control) represent the MF concentration and the sum of MF and FA concentrations in the sterile controls, respectively. Mean values of three replicates and standard deviations are shown

MF disappearance, FA accumulation, and the sum of MF and FA concentrations followed similar trends and magnitudes in incubations with MF and with Glc:MF (1:1) (Figure 2a,b). In the Glc:MF (13:1) treatment, the sum of MF and FA concentrations decreased sharply between 4 and 12 hr before slowing down during the remaining incubation period (Figure 2c).

**Figure 3:**
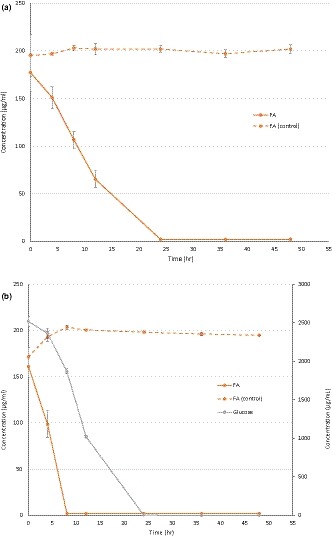
Ferulic acid (FA) disappearance during cultivation of *Lactobacillus buchneri* LN 4,017 with 177 µg/ml FA (a) and 2,515 µg/ml glucose and 161 µg/ml FA (b). FA (control) represents FA concentration in the sterile control. Mean values of three replicates and standard deviations are shown

MF is used as a model substrate to study FAE activity, with FA as a product of MF hydrolysis (Donaghy et al., 1998; Wang et al., 2016). The continuous decrease of the sum of MF and FA concentrations in incubations with MF and with Glc:MF indicates that FA released was further metabolized. The ability of *L. buchneri* to metabolize FA also in the presence of glucose was confirmed in our secondary experiment with FA (Figure 3). In line with our results, it was previously shown that other *Lactobacillus* spp. are also able to metabolize FA. *L. plantarum* metabolized FA to 4‐vinylguaiacol and hydroferulic acid, and *L. collinoides* metabolized FA to 4‐vinylguaiacol (Knockaert, Raes, Wille, Struijs, & Camp, 2012). The metabolism of FA by *L. buchneri* was slowed down after 12 hr of incubation in the highest Glc:MF treatment (Figure 2c), resulting in FA accumulation between 24 and 48 hr.

Similar growth of cultures in the basal medium, with MF or with FA (Figure 1), implies that despite metabolism of FA, the bacteria did not assimilate FA, similar to the observations of Knockaert et al. (2012) with *L. plantarum* and *L. collinoides*. Bacterial growth in these treatments was mainly supported by the nutrients present in the basal medium.

The similar profiles of MF metabolism in incubations with MF and with Glc:MF (1:1) (Figure 2a,b) indicate that the presence of glucose did not affect MF hydrolysis. MF metabolism was also continued at the high concentration of glucose (Figure 2c), indicating that the de‐esterification ability of *L. buchneri* was not repressed by glucose.

We made an estimation of the ratio of fermentable sugars to cell wall‐associated ester linkages in silage to examine reliability of our experimental setup. It should be noted that such ratio varies extensively from case to case as the sugar contents of forages and cell wall‐associated ester linkages vary by forage type, forage maturity, climate, etc. Following assumptions were made. (a) Concentration of water‐soluble carbohydrates (WSC) in silage crops is on average 15% of dry matter (DM), with glucose comprising 21% of WSC (Müller, Rosen, & Udén, 2008). (b) Neutral detergent fiber (Van Soest, Robertson, & Lewis, 1991), with an average concentration of 49% of DM (Müller et al., 2008), represents plant cell walls. (c) *Trans*‐FA of plant cell walls, with an average concentration of 0.54% of cell walls (Hartley & Jones, 1977), represents cell wall‐associated ester linkages. Under these conditions, the ratio of glucose:FA becomes 12:1 on mass basis, in agreement with our experimental setup.

The sterile controls were included to ensure that there was no abiotic degradation of MF and FA. There was an increase in the concentration of FA in the sterile control of Glc:FA treatment between 0 and 8 hr (Figure 3b), likely due to sampling/pipetting errors. As this increase did not interfere with data interpretation, it was ignored.

## 4 CONCLUSIONS

FA released from hydrolysis of MF was further metabolized by *L. buchneri* LN 4017 but did not support bacterial growth. MF hydrolysis was almost similar at all concentrations of glucose, indicating that the de‐esterification activity of *L. buchneri* LN 4017 was not repressed by glucose*.* We therefore suggest that de‐esterification activity of *L. buchneri* LN 4017, mediated by the action of FAE, is not repressed by substrates present in silage. Our results, however, should be complemented with transcriptomic/proteomic studies to provide firm conclusions.

## CONFLICT OF INTERESTS

None declared.

## AUTHORS CONTRIBUTION

All authors contributed to conceptualization of the experiment. Kamyar Mogodiniyai Kasmaei conducted the experiment and wrote the original draft. All authors contributed to final revision of the manuscript.

## ETHICAL APPROVAL

None required.

## Data Availability

The raw data supporting the conclusions of this manuscript will be made available on request by the authors, without undue reservation, to any qualified researcher.
